# Comparison of serum Lp‐PLA2 levels in ischemic stroke patients with H‐type hypertension or non‐H‐type hypertension

**DOI:** 10.1002/jcla.23068

**Published:** 2019-10-19

**Authors:** Jie Qiao, KuiChen Zhou, Chao Huang, Siwen Fu, Yonghong Xing, Biao Zhang

**Affiliations:** ^1^ Tianjin Key Laboratory of Cerebral Vessels and Neural Degeneration Tianjin Neurosurgical Institute Tianjin Huanhu Hospital Tianjin China; ^2^ Department of Clinical Laboratory The First Affiliated Hospital of Jiamusi University Jiamusi China

**Keywords:** homocyteine, H‐type hypertension, ischemic stroke, Lipoprotein‐associated phospholipase‐A2

## Abstract

**Background:**

Increased serum Lp‐PLA2 levels have been reported in patients who experienced an ischemic stroke; however, the relationship between Lp‐PLA2 and H‐type hypertension in patients with ischemic stroke remains unclear.

**Methods:**

In the present study, we investigated the correlation between serum Lp‐PLA2 and H‐type hypertension in patients with ischemic stroke. A total of 135 patients who experienced acute ischemic stroke were enrolled in Tianjin Huanhu Hospital during October 2015 to May 2016. The demographic characteristics of the patients were collected, and biochemical parameters were detected.

**Results:**

Among the 135 patients, 111 patients had essential hypertension, including 41 patients with H‐type hypertension and 70 with non‐H‐type hypertension. There were higher proportions of males and patients with diabetes mellitus in the H‐type hypertension group compared with the non‐H‐type hypertension group (*P* < .05). Lp‐PLA2, glucose, and LDL‐C levels were higher in the H‐type hypertension group than in the non‐H‐type hypertension group (*P* < .05). Multivariate logistic regression showed that Lp‐PLA2 levels >208.46 mg/L were independently associated with H‐type hypertension in patients with ischemic stroke (OR: 2.560, 95% CI: 1.085‐6.040, *P* = .032). The area under the ROC curve of Lp‐PAL2 levels in the H‐type hypertension group was 0.665 (95% CI: 0.561‐0.768, *P* = .004).

**Conclusion:**

Synergetic effects of H‐type hypertension and Lp‐PLA2 levels were noted in the pathogenesis of ischemic stroke.

## 1 INTRODUCTION

Ischemic stroke is one of the most frequently reported cerebrovascular diseases, and it has become the major cause of adult death and disability in China.^1^ Accumulating evidence has shown that various risk factors are associated with ischemic stroke, among which atherosclerosis, hypertension, and increased hyperhomocysteine levels are the most important factors.^2^, ^3^, ^4^ Atherosclerosis plaque is one of the pathological mechanisms of ischemic stroke. Presently, it is well established that atherosclerosis is a chronic inflammatory disease. Several inflammatory biomarkers are associated with atherosclerosis, and inflammatory biomarkers may reflect the development of atherosclerotic plaques.^5^, ^6^

Lipoprotein‐associated phospholipase A2 (Lp‐PLA2) is a new inflammatory biomarker, which is secreted by monocytes, macrophages, and T cells.^7^ High Lp‐PLA2 levels have been associated with an increased risk of atherosclerosis and ischemic stroke.^8^ In the blood circulation, Lp‐PLA2 primarily binds to LDL‐C and HDL‐C.^7^, ^9^ Lp‐PLA2 can generate the pro‐inflammatory molecules lysophosphatidylcholine and oxidized free fatty acids by hydrolyzing phospholipids of LDL‐C and HDL‐C, and these pro‐inflammatory molecules promote ischemic stroke.^10^

Essential hypertension increases the shear stress of blood fluids, which impairs the endothelium and aggravates vascular smooth muscle cell proliferation, leading to atherosclerosis formation. Hyperhomocysteine is another risk factor for ischemic stroke.^11^ Recently, the association between hypertension and hyperhomocysteine in the pathogenesis of ischemic stroke has received more attention. As risk factors, hypertension and hyperhomocysteine have a cumulative effect on the pathogenesis and development of ischemic stroke. Thus, the term H‐type hypertension was proposed, which was defined as hypertension together with hyperhomocysteine more than 10 μmol/L.^12^, ^13^ Numerous studies have found that H‐type hypertension increased the risk of ischemic stroke compared with non‐H‐type hypertension.^14^, ^15^ Although a relationship between Lp‐PLA2 level and ischemic stroke has been demonstrated by several studies,^8^, ^16^ the relationship between serum Lp‐PLA2 levels and H‐type hypertension in patients with ischemic stroke remains poorly understood.

The purpose of this investigation was to compare the Lp‐PLA2 level in patients with ischemic stroke with or without H‐type hypertension and to explore the correlation between Lp‐PLA2 and H‐type hypertension in patients with ischemic stroke.

## 2 MATERIALS AND METHODS

### 2.1 Patients

This investigation was approved by the ethics committee of Tianjin Huanhu Hospital. In this retrospective study, we consecutively recruited a total of 135 acute ischemic stroke (AIS) patients from Tianjin Huanhu Hospital from October 2015 to May 2016, including 45 females and 90 males with an age range of 31‐92 years. The inclusion criteria were as follows: patients were 18 years or older, patients were admitted within 24 hours after the time of symptom onset, and patients had hypertension. All patients were diagnosed by a neurologist based on a neurological examination and further confirmed using CT or MRI in accordance with the Chinese guidelines for the diagnosis and treatment of acute ischemic stroke 2014.^17^ Of 135 patients with AIS, 111 patients had essential hypertension and 24 patients with secondary hypertension were excluded. Seventy‐eight patients had large artery atherosclerosis, and 33 patients had small‐vessel occlusion. Sixty‐four healthy volunteers were recruited as controls.

### 2.2 Exclusion criteria

Exclusion criteria included transient ischemic attack, intracerebral hemorrhage, subarachnoid hemorrhage, malignant tumor, chemotherapy, severe liver and renal dysfunction, febrile disorders, severe edema, heart disease, systemic infections, autoimmune diseases, and secondary hypertension.

### 2.3 Demographic data collection

The general demographic characteristics of patients and controls were collected, including gender, age, smoking, alcohol consumption, body mass index, carotid atherosclerosis, diabetes mellitus, hypertension, and biochemical parameters.

### 2.4 Laboratory examination

Elbow venous blood samples were collected from all patients in the morning on day 1 of the hospital admission after overnight fasting. For biochemical tests, blood samples were injected into separation gel tubes. For fibrinogen detection (FIB), blood was drawn into a heparin anticoagulant tube and samples were immediately sent for analysis. Serum total cholesterol (TC), triglycerides (TG), LDL cholesterol (LDL‐C), HDL cholesterol (HDL‐C), and glucose were measured using a Beckman AU5800 automatic biochemistry analyzer. Plasma HCY and serum high sensitive C‐reactive protein (hs‐CRP) levels were determined using BNII immune turbidimetry, and fibrinogen was determined using Sysmex CS‐5100. The Lp‐PLA2 level was determined using the quantitative sandwich enzyme‐linked immunosorbent assay kit (Kangerke Bioscience) in accordance with the manufacturer's instructions.

### 2.5 Statistical analysis

Data were analyzed using SPSS software version 16.0. For continuous variables, data were presented as the median and range. For categorical variables, data were described as the frequencies and percentages. Student's *t* test was used for continuous variables and data with a normal distribution, and a Wilcoxon rank‐sum test was used for comparing the data that did not have a normal distribution. Chi‐squared test was used for comparing categorical variables. Univariate and multivariate logistic regression analyses were performed for identifying the risk factors for patients with ischemic stroke with H‐type hypertension. Multivariate logistic regression analysis was also used for determining the risk factors for patients with ischemic stroke with H‐type hypertension. Receiver operating characteristic (ROC) curve analysis was performed for determining the diagnostic properties of Lp‐PLA2 levels in the patients with ischemic stroke with H‐type hypertension as compared with those with non‐H‐type hypertension. All *P* values were two‐tailed and *P* < .05 was considered statistically significant.

## 3 RESULTS

### 3.1 Demographic characteristics and biochemical parameters of the patients and controls

The demographic characteristics and biochemical parameters of the patients with AIS and controls are shown in Table 1. In comparison with the controls, patients with AIS had more traditional risk factors. Briefly, patients with AIS had a higher prevalence of smoking and higher glucose, LDL‐C, FIB, and HCY levels. Moreover, serum Lp‐PLA2 levels were significantly higher in patients with AIS as compared with the controls. No significant difference was noted among other variables between patients with AIS and controls (*P* > .05).

**Table 1:** Demographic characteristics and biochemical parameters of the recruited subjects

Variable	Healthy control (n = 64)	IS patients with Hypertension (n = 111)	*P*
Gender (male/female), n/n	39/25	68/43	.546
Age (median, range), years	62.6 ± 8.24	65.2 ± 11.49	.115
BMI, kg/m^2^	25.00 ± 1.74	24.94 ± 1.36	.797
Diastolic blood pressure (mm Hg)	84 ± 16	95 ± 14	.000*
Systolic blood pressure (mm Hg)	137 ± 19	151 ± 23	.000*
Diabetes mellitus, n (%)	‐	41 (36.9)	‐
Dyslipidemia, n (%)	‐	58 (43.0)	‐
Carotid atherosclerosis, n (%)	‐	58 (43.0)	‐
Alcohol consumption, n (%)	30 (46.9)	52 (46.8)	1.000
Cigarette smoking, n (%)	50 (78.1)	100 (90.1)	.042*
NIHSS (median, range)	‐	5 (0‐23)	‐
HCY (median, range),mol/L	12.11 (6.83‐19.80)	13.64 (3.95‐69.90)	.012*
FIB (median, range),g/L	2.81 (2.14‐5.73)	3.10 (1.20‐6.99)	.038*
Glucose (median, range),mmol/L	5.10 (3.91‐6.73)	5.50 (2.80‐18.20)	.015*
TC(median, range), mmol/L	4.75 (3.04‐7.43)	5.35 (1.00‐9.93)	.011
TG (median, range), mmol/L	1.24(0.43‐3.81)	1.34 (0.48‐17.38)	.210
LDL‐C (median, range),	2.82 (2.14‐5.73)	2.96 (0.83‐7.35)	.001*
HDL‐C (median, range),	1.29 (0.71‐2.69)	1.08 (0.58‐2.84)	.000
Hs‐CRP (median, range),g/L	0.47 (0.16‐3.85)	2.46 (0.27‐178.00)	.000
Lp‐PLA2 (median, range),mg/L	141.60 (26.48‐281.30)	164.51 (40.44‐584.23)	.001*
Lp‐PLA2 > 208.46 mg/L, n (%)	8(12.50)	30 (27.03)	.026*

* *P* < .05.

### 3.2 Comparison of demographic characteristics and biochemical parameters between H‐type hypertension and non‐H‐type hypertension groups

The demographic characteristics and biochemical parameters of the patients with AIS are presented in Table 2. On the basis of the essential hypertension and homocysteine levels, 111 patients with AIS with essential hypertension, defined as systolic blood pressure ≥140 mm Hg and/or diastolic blood pressure ≥90 mm Hg, were divided into the H‐type hypertension (hemocysteine concentration ≥10 μmol/L) and non‐H‐type hypertension groups (hemocysteine concentration <10 μmol/L), according to previous report.^12^, ^13^ Forty‐one patients and 70 patients were included in the two groups, respectively.

**Table 2:** Comparison of demographic characteristics and biochemical parameters between H‐type hypertension and non‐H‐type hypertension patient groups

Variable	H‐type Hypertension (n = 41)	Non‐H‐type Hypertension (n = 70)	*P*
Gender (male/female), n/n	31/10	37/33	.014*
Age (median, range), years	67.1 ± 12.9	64.1 ± 10.5	.163
BMI, kg/m^2^	24.95 ± 1.34	24.93 ± 1.38	.927
Diastolic blood pressure (mm Hg)	95 ± 15	94 ± 14	.724
Systolic blood pressure (mm Hg)	151 ± 23	152 ± 22	.822
Diabetes mellitus, n (%)	22 (53.7)	19 (27.1)	.004*
Dyslipidemia, n (%)	24 (58.5)	34 (48.6)	.332
Carotid atherosclerosis, n (%)	25 (61.0)	33 (47.1)	.174
Alcohol consumption, n (%)	20 (48.7)	32 (45.7)	.341
Cigarette smoking, n (%)	36 (90.2)	61 (87.1)	.584
NIHSS (median, range)	4 (0‐17)	5 (0‐23)	.978
FIB (median, range),g/L	3.16(1.69‐4.82)	3.01(1.20‐6.99)	.376
Glucose (median, range),mmol/L	5.83(2.80‐18.20)	5.03(4.00‐10.53)	.010*
TC (median, range), mmol/L	4.83(2.93‐9.93)	4.98(1.00‐8.64)	.310
TG (median, range), mmol/L	1.34(0.52‐7.12)	1.36(0.48‐17.38)	.415
LDL‐C (median, range),	3.04( 1.26‐7.35)	2.96 (0.83‐5.44)	.043*
HDL‐C (median, range),	1.08 (0.68‐1.84)	1.08 (0.58‐2.84)	.517
Hs‐CRP (median, range), g/L	2.68 (0.37‐64.10)	2.45 (0.27‐178.00)	.891
Lp‐PLA2 (median, range),mg/L	191.55 (51.27‐387.16)	150.79 (40.44‐584.23)	.009*
Lp‐PLA2 > 208.46 mg/L,n (%)	16 (39.0)	14 (20.0)	.026*

* *P* < .05

**Figure 1:**
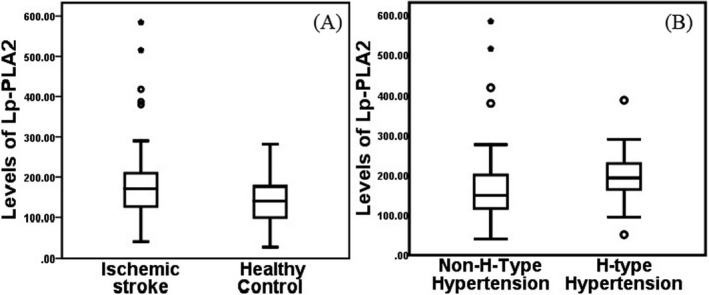
Levels of Lp‐PLA2 in healthy controls and patients with ischemic stroke. A: comparison of Lp‐PLA2 between healthy control and ischemic stroke; B: comparison of Lp‐PLA2 between H‐type hypertension group and non‐H‐type hypertension group

When compared with the non‐H‐type hypertension group, the patients in the H‐type hypertension group had increased proportions of males and patients with diabetes mellitus (*P* < .05; Table 2). The Lp‐PLA2, glucose, and LDL‐C levels were higher in the H‐type hypertension group compared with the non‐H‐type hypertension group (*P* < .05; Table 2, Figure 1), and the levels of other variables had no differences between the two groups (Table 2). The percentage of high Lp‐PLA2 levels (>208.46 mg/L) was increased in the H‐type hypertension group (*P* < .05). And there were no difference of biochemical parameters between males and females patients with ischemic stroke (Table 3).

**Table 3:** Comparison of biochemical parameters between males and females who were patients with AIS

Variable	AIS patients with Hypertension (n = 111)		*P*
	Male(n = 68)	Female(n = 43)	
HCY (median, range),mol/L	14.25 (3.95‐69.90)	12.60 (6.90‐46.30)	.062
FIB (median, range),g/L	3.12 (1.20‐6.99)	3.10 (1.62‐4.69)	.873
Glucose(median, range),mmol/L	5.30 (2.80‐15.12)	5.84 (4.07‐18.20)	.086
TC (median, range), mmol/L	4.72 (2.93‐9.93)	5.11(1.00‐8.42)	.650
TG (median, range), mmol/L	1.34 (0.48‐17.38)	1.34(0.65‐13.73)	.986
LDL‐C (median, range),	3.01 (0.83‐7.35)	2.98 (1.12‐5.44)	.517
HDL‐C (median, range),	1.02 (0.58‐1.91)	1.13 (0.74‐2.84)	.094
Hs‐CRP (median, range),g/L	2.74 (0.27‐178.00)	2.45 (0.27‐137.00)	.674
Lp‐PLA2 (median, range),mg/L	173.75 (51.27‐584.23)	170.52 (40.44‐418.52)	.582
Lp‐PLA2 > 208.46 mg/L,n (%)	20 (29.41)	10 (30.30)	.518

### 3.3 Risk factors for ischemic stroke patients with H‐type hypertension

Univariate and multivariate logistic regression analysis was used for identifying the risk factors for patients with ischemic stroke with H‐type hypertension. First, univariate logistic regression analysis was used for assessing the variables that had differences between the H‐type hypertension and non‐H‐type hypertension groups. Variables with *P* values < .1 were then included in the multivariate regression analysis. As shown in Table 4, the results of univariate logistic regression analysis showed that gender, diabetes mellitus, glucose levels, LDL‐C, Lp‐PLA2, and the upper quartile of Lp‐PLA2 concentrations (Lp‐PLA2 > 208.46 mg/L) were significantly associated with H‐type hypertension. The multivariate logistic regression results showed that a Lp‐PLA2 concentration >208.46 mg/L was finally included in the model as an independent variable associated with patients with ischemic stroke with H‐type hypertension (OR: 2.560, 95% CI: 1.085‐6.040, *P* = .032).

3.4

**Table 4:** Univariable and multivariable analyses of risk factors associated with patients with ischemic stroke with H‐type hypertension

Variables	Univariable			Multivariable		
	OR	95% CI	*P*	OR	95% CI	*P*
Gender	2.297	0.996‐5.297	.051	0.534	0.219‐1.299	.166
Diabetes mellitus	0.309	0.134‐0.712	.006*	0.479	0.151‐1.522	.212
Glucose	0.785	0.638‐0.965	.022*	0.864	0.668‐1.117	.265
LDL‐C	1.477	0.959‐2.275	.177			
Lp‐PLA2	1.003	0.999‐1.008	.166			
Lp‐PLA2 > 208.46	2.560	1.085‐6.040	.032*	2.560	1.085‐6.040	.032*

* *P* < .05.

### 3.5 ROC curve analysis

**Figure 2:**
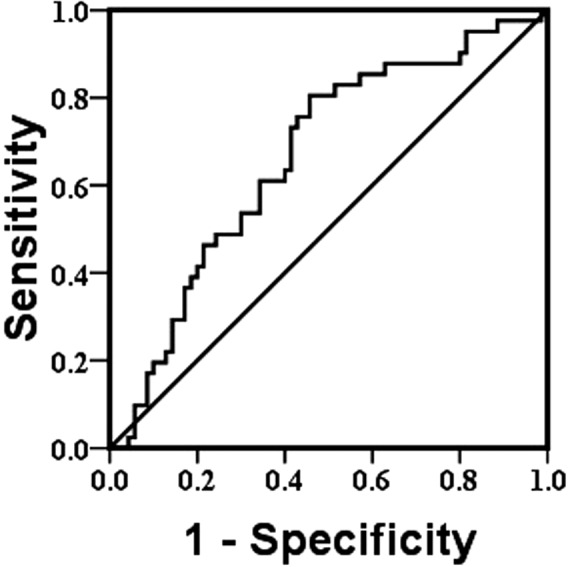
Receive operating characteristic (ROC) curve for Lp‐PLA2. Area under the ROC curve for Lp‐PLA2 was 0.665 with 95% CI: 0.561‐0.768, *P* = .004

To explore the capability of serum Lp‐PAL2 levels to identify patients with ischemic stroke with H‐type hypertension, ROC curve analysis was performed. The area under the ROC curve of Lp‐PAL2 levels for H‐type hypertension was 0.665 (95% CI: 0.561‐0.768, *P* = .004). The ROC curve showed that the optimal cutoff value for Lp‐PLA2 levels was 156.465 mg/L, and the sensitivity and specificity for the diagnosis of H‐type hypertension in ischemic stroke were 80.5% and 54.3%, respectively (Figure 2).

## 4 DISCUSSION

Ischemic stroke is a frequently occurring neurological disease with characteristics including high incidence, high recurrence, and high mortality rates. Ischemic stroke is a severe threat to people's health and life worldwide. Numerous reports have demonstrated that inflammatory markers were potential pathogenesis factors, and several biomarkers had been identified as predictors of outcomes for ischemic stroke, such as pro‐BNP, BNP, cortical, and copeptin, which were correlated with short‐term outcomes and mortality rates in AIS, as well as independent short‐term prognostic biomarkers of functional outcomes of AIS.^18^ Previous reports have demonstrated the association of hyperhomocysteine with ischemic stroke. Homocysteine may represent an important pathogenic factor in some types of stroke,^19^ and homocysteine causes inflammation and increases carotid artery atherosclerosis and formation of plaque, which is involved in multiple mechanisms, including effects on atrial ionic channels and the thrombotic state.^20^ Homocysteine levels, as an independent risk factor, may favor an ischemic stroke by promoting arterial stiffness,^21^, ^22^ and may determine more subsequent and severe complications.^23^ Hypertension is a major risk factor for ischemic stroke, which, combined with hyperhomocysteine, may increase the incidence of ischemic stroke.^24^ A recent study showed that Lp‐PLA2 can serve as a new inflammation marker associated with ischemic stroke. It may be directly involved in atherosclerosis, and it is able to independently forecast cerebrovascular events.^25^, ^26^

In the present study, we observed that serum Lp‐LPA2 levels were significantly increased in patients who had an ischemic stroke with H‐type hypertension compared with those with non‐H‐type hypertension. The percentages of high Lp‐PLA2 values (>208.46 mg/L) in the H‐type hypertension group were higher than those in the non‐H‐type hypertension group after adjusting for significantly different risk factors between the two groups, such as gender, diabetes, glucose, and HDL‐C, and higher Lp‐LPA2 levels were independent risk factors for the incidence of ischemic stroke in patients with H‐type hypertension. Patients with H‐type hypertension with Lp‐LPA2 concentration more than 208.46 mg/L were associated with a 2.56‐fold increase to have an ischemic stroke compared with patients with non‐H‐type hypertension. Moreover, ROC curve analysis indicated that Lp‐PLA2 may be a favorable diagnosis for ischemic stroke in patients with H‐type hypertension. These data emphasized the increase in ischemic stroke risk among patients with H‐hypertension who had high Lp‐PLA2 levels and demonstrated the potential application of Lp‐PLA2 in further characterizing ischemic stroke in patients with H‐type hypertension.

To the best of our knowledge, a few of the previous studies have investigated the relationship between Lp‐PLA2 levels and H‐type hypertension in ischemic stroke. Lp‐PLA2 is a biomarker of inflammation that exists in atherosclerotic plaques as well as in the bloodstream, and it binds to LDL‐C and HDL‐C.^7^, ^9^, ^10^ Lp‐PLA2 within plaques participated in inflammation and increased the rupture of plaques.^27^ A novel finding of the present study was that patients with ischemic stroke with H‐type hypertension had higher Lp‐PLA2 levels. High Lp‐PLA2 levels may collaborate with hyperhomocysteine to promote the incidence of ischemic stroke in patients with hypertension.

Hs‐CRP is a classic risk factor for inflammation associated with atherosclerosis, and the hs‐CRP levels are known to be significantly higher in patients who had an ischemic stroke than in controls. Acampa^28^ reported that hs‐CRP levels were associated with atrial fibrillation development in patients with cryptogenic stroke, and hs‐CRP may be a potential therapeutic target for preventing atrial fibrillation in patients with cryptogenic stroke. However, in this study, no significant difference was noted between ischemic stroke patients with H‐type hypertension and those with non‐H‐type hypertension. Thus, Lp‐PLA2 may be a more useful biomarker than hs‐CRP for evaluating the prognosis of ischemic stroke in patients with H‐type hypertension.

There were several limitations in the present investigation. First, the cohort in this study was relatively small, and additional large cohort studies are needed to further examine the association of Lp‐PLA2 with H‐type hypertension in patients with ischemic stroke. Second, this study missed the information on severity and recovery rates. Third, this study was a retrospective analysis. The sequential Lp‐PLA2 levels in the same patients could not be obtained, and causality could not be derived accurately. Fourth, the statistical methods also affected the results as indicated by Ahad.^29^ The findings of our study need to be verified with advanced statistical methods on the basis of larger study samples.

## 5 CONCLUSIONS

In conclusion, the present study showed Lp‐PLA2 levels were independently associated with H‐type hypertension in ischemic stroke, and Lp‐PLA2 synergized with H‐type hypertension to increase the risk of ischemic stroke, which may be associated with increasing inflammation reactions in atherosclerosis plaques. Further prospective studies, including large sample studies, are necessary to verify the results of this study.

## AUTHORS CONTRIBUTIONS

JQ: involved in writing—original draft; KZ and CH: involved in data curation; YX: involved in project administration; SF: involved in resources; YX and BZ: involved in funding acquisition. All authors approved the final manuscript.
